# The effect of telemedicine on secondary prevention of atherosclerotic cardiovascular disease: A systematic review and meta-analysis

**DOI:** 10.3389/fcvm.2022.1020744

**Published:** 2022-11-03

**Authors:** Liangying Deng, Qing Wu, Feng Ding, Yanfeng Liu, Jianping Shen, Yan Lin, Kaihu Shi, Bailin Zeng, Lixing Wu, Huangjin Tong

**Affiliations:** ^1^Affiliated Hospital of Integrated Traditional Chinese and Western Medicine, Nanjing University of Chinese Medicine, Nanjing, China; ^2^Nanjing Lishui District Hospital of Traditional Chinese Medicine, Nanjing, China; ^3^School of Basic Medicine and Clinical Pharmacy, China Pharmaceutical University, Nanjing, China

**Keywords:** telemedicine, secondary prevention, atherosclerotic cardiovascular disease (ASCVD), effect, systematic review, meta-analysis

## Abstract

**Aim:**

The purpose of this systematic review was to evaluate the efficiency of telemedicine on the secondary level of prevention of patients with arteriosclerotic cardiovascular disease (ASCVD), provide evidence for the application of telemedicine in secondary prevention and promote the development of telemedicine in secondary prevention.

**Methods:**

A computer-based search was conducted in MEDLINE, Embase, Pubmed, EBSCO, CINAHL, the Cochrane Library, and Web of Science. Randomized controlled trials regarding the effect of telemedicine on secondary prevention of ASCVD were included from inception to May, 2022. Meta-analysis was used to compare the results of the included studies by RevMan5.4 software. The Cochrane Collaboration bias risk tool was used to perform risk of bias assessment in this study. Outcomes included risk factors, physical activity and exercise, muscle function, exercise compliance, medication adherence, healthy diet, depression and anxiety, self-efficacy, knowledge score, economy, and safety endpoints. Subgroup analysis was carried out for different main intervention measures included in the literature.

**Results:**

A total of 32 randomized clinical studies (*n* = 10 997 participants) were included in the meta-analysis. Compared with usual secondary prevention (USP) group, participants in telemedicine of secondary prevention (TOSP) group showed significant improvement in some risk factors including BMI (MD –0.87, *p* = 0.002), SBP (MD –4.09, *p* = 0.007) and DBP (MD –2.91, *p* = 0.0002) when they use the telephone as the intervention. In physical activity and exercise, Patients in TOSP showed an improvement in VO2 Peak (mL⋅kg^–1^⋅min^–1^) (OR 1.58, *p* = 0.02), 6MWT (MD 21.41, *p* = 0.001), GSLTPA score (MD 2.89, *p* = 0.005). Effects on medication adherence, exercise compliance, muscle function, healthy diet, economy and self-efficacy were synthesized narratively. Patients in TOSP did not show a reduction in knowledge score, depression, anxiety and safety endpoints.

**Conclusion:**

There is a net benefit of secondary prevention supported by telemedicine (especially when using the telephone as an intervention) in patients with ASCVD in the terms of some risk factors, physical activity and exercise. There are still controversies in the improvement of medication adherence, exercise compliance, muscle function, healthy diet, knowledge score, self-efficacy and economy *via* telemedicine, which is worth exploring. Larger samples size and longer-term follow-ups are needed in future studies.

**Systematic review registration:**

[https://www.crd.york.ac.uk/PROSPERO/display_record.php?RecordID=330478], identifier [CRD42022330478].

## Introduction

Although the morbidity and mortality of atherosclerotic cardiovascular disease (ASCVD) are declining in many developed countries, it remains a leading cause of incidence and mortality (1). ASCVD caused by plaque buildup in arterial walls is a series of circulatory system diseases referring to the following conditions: coronary heart disease (CHD), cerebrovascular disease, and peripheral arterial disease (PAD) (2, 3). Nowadays several studies have indicated that the application of secondary cardiovascular prevention plays an important role in the intervention of ASCVD and its complications (4–7). The main interventions of secondary prevention include lifestyle modifications; lipid-lowering therapy; control of risk factors and antiplatelet therapy (8, 9). Efficient secondary prevention can reduce the recurrence of ASCVD and decrease mortality. The United Nations emphasizes secondary prevention of cardiovascular disease (CVD) as an important public priority. Besides, the United Nations has declared to adopt a global “25 × 25” goal to reduce by 25% premature (< 70 years) mortality from cancer, CVD, chronic respiratory disease, and diabetes by 2025. The designated action plan includes taking measures to reduce modifiable cardiovascular risk factors, improving the availability of drug therapy and counseling to prevent CVD, and increasing access to affordable fundamental technologies and essential drugs to manage non-communicable diseases (10).

The main risk factors have been identified for ASCVD in the past several decades, including hypertension, smoking, dyslipidemia, obesity, diabetes and so on (2, 11, 12). Nowadays risk factors could be treated effectively and safely, and most medicines are now easy to access and inexpensive. However, a high percentage of people experience an unhealthy lifestyle, and even in the high (residual) CVD risk population, few people are treated properly in the field of risk factors intervention (1).

Although it is important to carry out secondary prevention, it has been shown that the use of secondary prevention of ASCVD is insufficient for patients (6, 13, 14). Especially in the COVID-19 setting, there are likely to be potential implications for the patients with ASCVD to access secondary prevention measures. The pandemic leads to sick people being absent from in-person events in hospitals and rehabilitation centers (9). At the same time, medical resources become unavailable for patients needing secondary cardiovascular prevention due to COVID-19 (15). Enhanced systems of telemedicine and other remote support could provide medical services for patients with CVD and moderate the influence of the COVID-19 pandemic (16). The development of telemedicine has been kicked into high gear. Furthermore, the break of the COVID-19 pandemic has accelerated its use to reduce risk factors and support in the field of physical, psychological, and social wellbeing (17). Telemedicine, and eHealth, provide medical services *via* information and telecommunication technologies, which is a promising approach (18, 19). These interventions in telemedicine vary from individual to population levels, including text messages, phone calls, webpage, wearable devices, and mobile devices. Nevertheless, it is still unclear whether telemedicine can improve the efficient effect of secondary prevention in patients with ASCVD. To further address this issue, this study systematically reviewed the studies on the impact of telemedicine on the secondary prevention of patients with ASCVD.

## Materials and methods

### Protocol and registration

The study was conducted according to the Preferred Reporting Items for Systematic Reviews and Meta-Analysis (PRISMA) statement (20). The protocol of this systematic review and meta-analysis was prospectively registered at the International Prospective Register of Systematic Reviews (PROSPERO) (Prospero registration number: CRD42022330478).

### Inclusion and exclusion criteria

#### Participants

The included participants were adults with ASCVD aged 18 or older, who met the diagnostic criteria for (1) CHD, (2) myocardial infarction (MI), (3) acute coronary syndrome (ACS), (4) cerebral arterial thrombosis, (5) PAD or patients who have received arterial revascularization, including coronary intervention and coronary artery bypass surgery. Patients who are diagnosed with other diseases such as heart failure will not be included.

#### Intervention

Telemedicine of secondary prevention (TOSP) group: participants received remote intervention by investigators through mobile devices, including text messages, phone calls, mobile applications, mobile websites, e-mails, or other remote monitoring.

Usual secondary prevention (USP) group: participants received usual care or secondary prevention treatment without telemedicine intervention. There is no difference between the interventions of the trial group and the control group except for the remote intervention. The studies will be excluded If the intervention is unclear in studies.

#### Type of study

Only the randomized controlled trial was eligible as it is the most appropriate design for examining the efficient effect of telemedicine on the secondary prevention of ASCVD. We have conducted a pilot search and the result shows that a considerable number of randomized controlled trials were published. The studies were published in English.

#### Outcomes

Included studies should at least include one of the following outcomes: (1) primary outcomes: cardiovascular risk factor control; (2) secondary outcomes: physical activity and exercise, muscle function, exercise compliance, medication adherence, and healthy diet; (3) tertiary outcomes: depression and anxiety, self-efficacy, knowledge score, economy, safety endpoints, We extensively included all telemedicine studies on secondary prevention in patients with ASCVD in order to comprehensively analyze the effects of secondary prevention for patients with ASCVD.

### Search strategy

We searched seven databases: PubMed, Web of Science, CINAHL, EBSCO, MEDLINE, Embase, and Cochrane Library (May 2022) updating from the construction of the bank to May 2022. The search strategy is presented in Supplementary Table 1.

### Studies selection and quality evaluation

Two investigators (FD and QW) independently conducted the screening of studies according to the inclusion and exclusion criteria. Inconsistent or uncertain opinions of the included studies were judged by a third person (LYD).

We used the “risk of bias” according to the Cochrane Handbook for the quality evaluation of the included literature. Risks of bias were independently assessed for each study by two of the researchers (FD and QW) using the Cochrane Risk of Bias Assessment Tool, and the third review author (LYD) checked them for assessing the risk of bias including selection bias, performance bias, detection bias, attrition bias, reporting bias and other bias. The risk of bias for each outcome was assessed as low (green), unclear (yellow), or high (red).

### Statistical analysis

All calculations for the meta-analysis were conducted by Review Manager 5.4. For studies that measured outcomes at multiple time points, we decided to include outcomes with the longest follow-up after intervention in the meta-analysis due to wide variation in time points across studies. The mean difference (MD) with 95% CI was calculated for continuous data. Standardized mean difference (SMD) was used when varying outcome measurement instruments were used. The Risk Ratio (RR) and 95% confidence intervals (CI) were calculated for dichotomous data. Heterogeneity between studies was explored by Cochran’s Q statistic and *I*^2^ index. We adopted a random-effects model to perform the meta-analysis if *p* ≤ 0.1 or *I*^2^ > 50%; otherwise, a fixed-effects model was used. A random-effects model was used If there was no heterogeneity (*I*^2^ ≤ 50%, *p* > 0.1), the weights varied considerably between included studies and the number of studies was no higher than 3. Subgroup analysis was performed for our primary outcomes for our meta-analysis containing more than 10 studies according to the type of main intervention measures. The results are presented as forest plots with a 95% CI, with *p* < 0.05 deemed to be indicative of a statistically significant difference. In studies where median and interquartile ranges were reported, mean and standard deviations were estimated through methods recommended by Wan et al. (21). Studies that could not be analyzed for meta-analysis were using narrative synthesis instead. Sensitivity analysis was carried out by excluding each study one by one. We explored publication bias using funnel plots when the outcomes contain more than 10 studies.

## Results

### Search results

**FIGURE 1:**
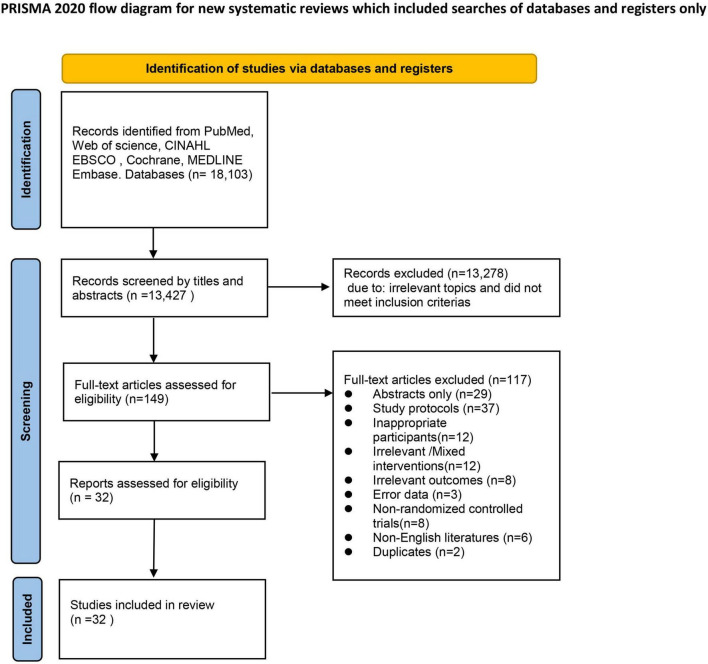
PRISMA flow diagram of study selection.

We identified 18,103 records from the initial search in the seven electronic databases and removed 4,676 duplicates. 13,278 records that were irrelevant and not met the inclusion criteria were excluded after the title and abstracts of 13,427 records were screened. We further conducted a full-text review of the remaining 149 studies and confirm whether they conform to eligibility criteria. At last, we included 32 items of studies for our systematic review and 25 items of studies for our meta-analysis (Figure 1).

### Characteristics of included studies

The 32 studies included 10,997 participants for analysis. We presented their main characteristics in Table 1. These studies were conducted in North America (*n* = 5) (22–26), Europe (*n* = 10) (14, 27–35), Asia (*n* = 9) (19, 36–43), Africa (*n* = 1) (44), Australia and New Zealand (*n* = 6) (45–50), South America (*n* = 1) (13). We included participants who were diagnosed with MI (24, 29, 32, 34, 35, 37, 44, 47), ACS (14, 22, 31, 33, 49), CHD (13, 19, 23, 27, 28, 30, 40, 41, 45, 46, 48, 50), Ischemic stroke (13, 30) and PAD (13, 19, 30) or received coronary revascularization (13, 19, 23, 25, 26, 28, 29, 33, 36, 38, 39, 42, 43, 45, 48, 49) in our review. The participants’ mean age varies from 55.8 to 70.36 and 20.04% of them are female. Due to the existence of mixed intervention measures, we assume the main intervention measures as the measures of contact and interaction between researchers and patients. We classified the main intervention measures as telephone (14, 26, 28, 33, 35, 37, 39, 47, 48), e-mail and telephone (27), text message (22, 24, 31, 36, 49, 50), mobile application (13, 19, 32, 38, 42–46), website (23, 25, 29, 30, 34, 41) and website and mobile application (40).

**TABLE 1:** Characteristics of included studies.

First author (year)/ country	Population (intervention) a. Number (N) b. Age [Mean (SD)] c. Gender female (%)	Population (control) a. Number (N) b. Age [Mean (SD)] c. Gender female (%)	Diagnosis	Main telemedicine technology	Description of intervention	Follow-up time	Outcomes a. Factor risks b. Lifestyle c. Medicine d. Economics e. Others outcomes
Avilai et al. (27)/Belgium	Intervention: a. *N* = 26 b. 62.2 (7.1) c. 12%	Control: a. *N* = 25 b. 63.7 (7.4) c. 8%	CHD	Telephone/e-mail	A mobile application was used by patients to log their exercise data and upload the data for review by the investigators. Patients received feedback once a week by phone or e-mail.	12 months	a. BMI, LDL-C, SBP/DBP, fasting glucose b. Peak HR, Peak RER RPE, VO_2_ Peak, VT1, VT2, strength function
Bae et al. (36)/Korea	Intervention: a. *N* = 377 b. 60.1 (10.6) c. 16.3%	Control: a. *N* = 349 b. 60.7 (10.4) c. 17%	CHD or post-PCI	Text message	Access to a supporting website and SMS text messages regarding lifestyle modification were provided to the intervention group.	6 months	a. BMI, LDL-C, SBP/DBP
Batalik et al. (28)/Czech republic	Intervention: a. *N* = 23 b. 56.1 (6.8) c. 26%	Control: a. *N* = 21 b. 57.1 (7.9) c. 10%	CHD	Telephone	A telephone consultation received by each patient based on the telemonitoring and the physiotherapist gave telephone feedback once a week	12 months	a. BMI, b. Peak HR, Peak RER, VO_2_ peak, workload peak
Bermon et al. (13)/Colombia	Intervention: a. *N* = 391 b. 64.0 (9.7) c. 23.6%	Control: a. *N* = 414 b. 63.1 (10) c. 19.7%	CHD or ischemic cerebrovascular disease or peripheral arterial disease or coronary revascularization	Mobile application	The messages received by the participants including information on the health implications of adherence to healthy habits and indications and recommendations on how to take their medication and promote healthy medication habits	12 months	e. all-cause hospitalization, all-cause mortality, cardiac mortality, cardiac hospitalization
Brouwers et al. (45)/ Netherlands	Intervention: a. *N* = 153	Control: a. *N* = 147	CHD or coronary revascularization	Mobile application	A web-based application used by patients to upload the sensor data at least once per week and reviewed these data during video consultations.	12 months	d. cardiac health care total costs,
Bruggmann et al. (29)/Switzerland	Intervention: a. *N* = 38 b. 56.20 (5.15) c. 18%	Control: a. *N* = 30 b. 63 (11.68) c. 11%	Post-PCI with STEMT or NSTEMT	Website	Patients had watched the video on the e-learning website. Questions-answering and distribution of study questionnaires were conducted by the pharmacist.	6 months	e. knowledge scores
Chan et al. (37)/Singapore	Intervention: a. *N* = 130 b. 55.3 (8.5) c. 5.4%	Control: a. *N* = 124 b. 54.7 (9.1) c. 5.3%	STEMI or NSTEMI	Telephone	Using feed-forward blood pressure monitoring, app-based education, medication reminders, and remote consultations, the telehealth group was monitored and managed on medication adherence, dosage titration, and drug side effects.	12 months	d.all-cause hospitalization, cardiac hospitalization
Foccardi et al. (38)/Italy	Intervention: a. *N* = 15 b. 61.4 (8.9) c. 12.5%	Control: a. *N* = 15 b. 61.1 (10.6) c. 81.3%	Coronary revascularization	Mobile application	Participants received a written exercise prescription and standardized text message for the follow-up period by smartphone app in each study	3 months	b. strength testing
Pandey et al. (24)/Canada	Intervention: a. *N* = 42 b. 64.6 (11.5) c. 50%	Control: a. *N* = 41 b. 62.1 (11.0) c. 39.02%	MI	Text messages	Participants received text messages to improve medication adherence and exercise	12 months	b. METS, mean days of exercise per month during follow-up c. percentage of days covered by medicine
Henriksson et al. (14)/Sweden	Intervention: a. *N* = 406 b. 67.3 (10.7) c. 27.6%	Control: a. *N* = 391 b. 68.4 (10.9) c. 29.7%	ACS	Telephone1	During the follow-up (telephone-based), participants were counseled on the importance of medication adherence, physical activity, exercise, and smoking cessation.	36 months	a. SBP/DBP, LDL-C
Kamel et al. (44)/Egypt	Intervention: a. *N* = 100 b. 56.2 (9.3) c. 27%	Control: a. *N* = 100 b. 55.8 (11.2) c. 30%	Acute STEMI	Mobile application	The patients were contacted to schedule free-of-charge teleconsultations *via* videoconferencing using the Zoom application starting. Every patient was arranged for at least one virtual visit per month for the 3-month follow-up period	4 months	a. smoking cessation rates
Kang et al. (19)/Korea	Intervention: a. *N* = 322 b. 57.4 (7.7) c. 16.2%	Control: a. *N* = 321 b. 59.2 (7.6) c. 18.6%	CHD or coronary revascularization or ischemic stroke or peripheral arterial disease	Mobile application	The mobile application provided the management of the secondary prevention of cardiovascular disease for participants	6 months	a. BMI, SBP/DBP, LDL-C, smoking cessation rates b. GSLTPA score e. PHQ-9,
Maddison et al. (49)/ New Zealand	Intervention: a. *N* = 153 b. 61 (11) c. 19.6%	Control: a. *N* = 153 b. 61 (11) c. 26.2%	ACS or coronary revascularization	Text message	A minimum of 1 core heart message was sent to the participants per day for 24 weeks. The message comprises core heart health content including education and support for taking medication regularly, eating a healthy diet, managing stress, and exercising regularly	24 weeks	b. fruit and vegetable guidelines
Ögmundsdóttir Michelsen et al. (32)/Sweden	Intervention: a. *N* = 100 b. 60.0 (8.9) c. 16%	Control: a. *N* = 49 b. 61.1 (8.6) c. 27%	AMI	Mobile application	A mobile application supported adherence to lifestyle advice and self-control of risk factors	14 months	a. BMI, SBP/DBP, fasting glucose, HbA1c (%), LDL-C b. healthy diet index, exercise capacity
Su and Yu (40)/China	Intervention: a. *N* = 73 b. 55.53 (7.30) c. 15.1%	Control: a. *N* = 73 b. 56.03 (7.02) c. 17.8%	CHD	Website and mobile application	Participants enhanced behavioral risk factor modification *via* the website and Mobile application	12 weeks	a. BMI, SBP/DBP e. DASS-21, CSES
Ross et al. (22)/Canada	Intervention: a. *N* = 38 b. 59.5 (9.1) c. 27%	Control: a. *N* = 38 b. 61.1 (9.6) c. 26%	ACS	Text message	The patients received a 60-day SMS text messaging intervention, covering a range of topics from time-sensitive information regarding their recovery to general healthy living advice	2 months	e. CSES, all-cause hospitalization, cardiac hospitalization
Snoek et al. (33)/Netherlands	Intervention: a. *N* = 61 b. 60.0 (8.4) c. 18%	Control: a. *N* = 61 b. 59.0 (10.7) c. 18%	ACS or coronary revascularization	Telephone	Participants were contacted every month by telephone, covering a range of topics (fitness, quality of life, cardiovascular risk factors, and care utilization)	52 weeks	a. BMI, SBP/DBP, LDL-C b. peak HR, peak RER, VO_2_ peak, workload peak, RPE (Borg) e. HADS, PHQ-9, MACE
Treskes et al. (34)/Netherland	Intervention: a. *N* = 100 b.60.03 (2.72) c.19%	Control: a. *N* = 100 b. 59.06 (8.95) c. 25%	AMI	Website	Participants received an electronic visit to a website	12 months	e. all-cause mortality, MACE
Wong et al. (41)/China	Intervention: a. *N* = 219 b. 52.22 (5.07) c. 32.42%	Control: a. *N* = 219 b. 52.46 (4.72) c. 36.62%	CHD	Website	Web-based educational support intervention was provided for the participants on total exercise and cardiovascular risk markers	6 months	a. SBP/DBP, LDL-C b. GSLTPA score, SEE-C
Yu et al. (42)/ China	Intervention: a. *N* = 501 b. 57.41 (8.99) c. 13.6%	Control: a. *N* = 499 b. 57.1 (9.20) c. 15.4%	Post-CABG	Mobile application	Smartphone-based application improved medication adherence	6 months	a. BMI
Hu et al. (39)/ China	Intervention: a. *N* = 420 b. 67.7 (4.65) c. 34.73%	Control: a. *N* = 420 b. 68.1 (4.23) c. 32.42%	Post-PCI	Telephone	The patients were provided cardiovascular risk management through an intensive nursing care electronic follow-up system	12 months	a. BMI, SBP, LDL-C b. Dietary control
Yudi et al. (46)/Australia	Intervention: a. *N* = 103 b. 56.8 (9.9) c. 14.5%	Control: a. *N* = 103 b. 56.2 (10.2) c. 16.7%	CHD	Mobile application	The app provided a platform to deliver a comprehensive secondary prevention program, including dynamic tracking of cardiovascular risk factors, exercise prescription, heart health education, education on secondary prevention pharmaco-therapy as well as feedback and support.	8 weeks	a. BMI, SBP/DBP, LDL-C, HbA1c (%), smoking cessation rates b. HADS, 6 min walk test
Pfaeffli Dale et al. (50)/ New Zealand	Intervention: a. *N* = 61 b. 59.0 (10.5) c. 21%	Control: a. *N* = 62 b. 59.9 (11.8) c. 16%	CHD	Text messages	The intervention group received a 24-week mHealth program sent by automated daily text messages and access to a supporting website commencing, improving adherence to recommended lifestyle behaviors.	6 months	a. BMI, SBP/DBP LDL-C, smoking cessation rates b. fruit and vegetable intake, physically active e. HADS, overall self-efficacy
Lear et al. (23)/Canada	Intervention: a. *N* = 38 b. 59.24 (10.70) c. 10%	Control: a. *N* = 40 b. 58.6(9.15) c. 20%	ACS or coronary revascularization	Website	Participants received physiologic data capture; heart rate monitoring; education sessions and ask-an-expert sessions *via* the website	12 months	c. self-report of medicine adherence
Dorje et al. (43)/China	Intervention: a. *N* = 156 b. 59.1 (9.4) c. 18%	Control: a. *N* = 156 b. 61.9 (8.7) c. 19%	Post-PCI	Mobile application	Participants received a smartphone- based cardiac rehabilitation and secondary prevention programs delivered *via* the social media	12 months	a. BMI, SBP, LDL-C b. 6 min walk test (m) e. knowledge of CHD total score, GAD-7 total score, PHQ-9
Greving et al. (30)/ Netherlands	Intervention: a. *N* = 164 b. 60.7 (7.8) c. 22%	Control: a. *N* = 166 b. 59.2 (8.9) c. 29%	Atherosclerosis in the coronary, cerebral, or peripheral arteries	Website	Participants received self-management support, monitoring of disease control, and drug treatment *via* the website with a nurse practitioner for 12 months	12 months	a. BMI, LDL-C, SBP/DBP, fasting glucose, HbA1c (%), smoking cessation rates d. EQ-5D[QALY] e. MACE
Blasco et al. (31)/Spain	Intervention: a. *N* = 102 b. 60.6 (11.5) c. 18.6%	Control: a. *N* = 101 b. 61 (12.1) c. 20.8%	ACS	Text messages	Weight, heart rate, blood pressure (BP), capillary plasma lipid profile and glucose monthly were sent by patients using mobile phones weekly. A cardiologist accessed these data through a web interface and sent recommendations *via* messages service.	12 months	a. BMI, SBP/DBP
Hanssen et al. (35)/Norway	Intervention: a. *N* = 156 b. 59.5 (12.9) c. 15.4%	Control: a. *N* = 132 b. 60.9 (10.8) c. 23.5%	AMI	Telephone	Information and support were provided by a nurse to patients who had acute myocardial infarction by telephone	6 months	a. smoking cessation rates b. How often on average are you exercising each week
O’Neil et al. (47)/Australia	Intervention: a. *N* = 141 b. 62 (11.0) c. 21.3%	Control: a. *N* = 156 b. 59.7 (10.4) c. 20.5%	MI	Telephone	The health coach improved psychological outcomes *via* telephone	6 months	e. HADS
Reid et al. (25)/Canada	Intervention: a. *N* = 115 b. 56.7 (9.0) c. 17.4%	Control: a. *N* = 108 b. 56.0 (9.0) c. 13.9%	Post-PCI (ACS)	Website	Participants completed five online tutorials over 6 months and were contacted by e-mail by an exercise specialist. Physical activity was measured by pedometer and self-reported	12 months	b. Physical activity (minutes/week)
Smith et al. (26)/America	Intervention: a. *N* = 70 b. 70.26 (10.7) c. 12.9%	Control: a. *N* = 70 b. 70.36 (8.26) c. 20.3%	Post-CABG	Telephone	Participants received exercise capacity and habitual physical activity *via* telephone-monitored home-based exercise training during cardiac rehabilitation	6 years	a. BMI b. VO_2_ Peak (ml/min) e. all-cause hospitalization,
Vale et al. (48)/Australia	Intervention: a. *N* = 398 b. 58.6 (10.6) c. 21%	Control: a. *N* = 392 b. 58.3 (10.6) c. 25%	CHD or coronary revascularization	Telephone	Participants were provided monitoring of disease control, self-management support, and drug treatment through website and email	6 months	b. total fat, fiber

ASCVD, atherosclerotic cardiovascular disease; CHD, coronary heart disease; PCI, percutaneous coronary intervention; STEMI, ST-segment elevation myocardial; AMI, acute myocardial infarction; MI, myocardial infarction, NSTEMI, non-ST-segment elevation myocardial infarction; ACS, acute Coronary Syndromes; CABG, coronary artery bypass graft; Coronary revascularization included PCI and CABG; BMI, body Mass Index; LDL-C, low-density lipoprotein; SBP, systolic blood pressure; DBP, diastolic blood pressure; HADS, the hospital anxiety and depression scale; PHQ-9, the patient health questionnaire; DASS-21, depression anxiety and stress scale; GAD-7, Generalized Anxiety Disorder 7-item; MACE, major adverse cardiovascular event; EQ-5D (QALY), EuroQol five dimensions questionnaire (Quality-adjusted life year); RER, respiratory exchange ratio; RPE(Borg), rating of perceived exertion (borg); Peak HR, peak heart rate; Peak VO_2_, peak oxygen consumption, VT1, first ventilatory threshold; VT2, second ventilatory threshold; CSES; the Cardiac Self-efficacy Scale; METS, metabolic equivalents, GSLTPA, godin-sheppard leisure-time physical activity questionnaire; SEE-C, the Self-Efficacy for Exercise Scale.

### Included studies quality evaluation

**FIGURE 2:**
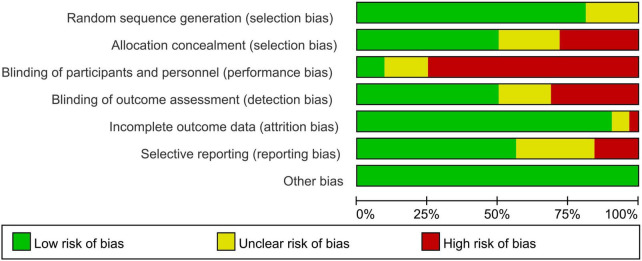
Risk of bias assessment included studies.

26 studies of the 32 included RCTs were found to be clearly detailed in random sequence generation. Unclear risk of selection bias existed in 7 studies for not describing the way allocation concealment was undertaken. It is impossible to blind participants and personnel in all studies because of the characteristics of the intervention. Therefore, performance bias cannot be avoided to have a high risk. In our included studies, there are 24 studies in which blinding research staff and participants have not been carried out. In 16 studies, the outcome assessors were blinded to avoid detection bias. Twenty-nine studies showed that the low risk of attrition bias was due to either the study’s appropriate solution for missing data or no significant differences in dropouts between the two groups. 18 studies had a protocol and reported predicted outcome indicators according to the protocol and were considered to have a low risk of reporting bias. There is no other bias. A summary of the results of the risk of bias was presented in Supplementary Figure 1. The risk of bias assessment included studies was presented in Figure 2.

### Outcomes

#### Primary outcomes

##### Risk factors

The primary outcomes included modifiable risk factors body mass index (BMI), systolic blood pressure (SBP), diastolic blood pressure (DBP), low-density lipoprotein cholesterol (LDL-C), fasting glucose, glycosylated hemoglobin (HbA1c), and smoking cessation rates.

**FIGURE 3:**
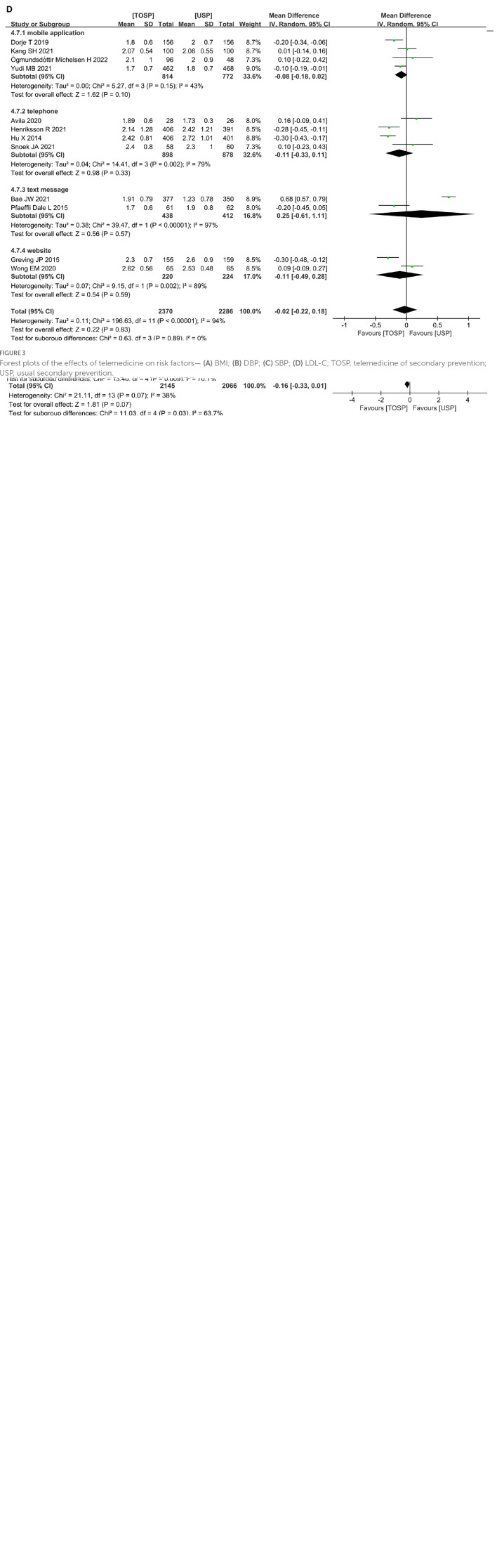
Forest plots of the effects of telemedicine on risk factors— **(A)** BMI; **(B)** DBP; **(C)** SBP; **(D)** LDL-C; TOSP, telemedicine of secondary prevention; USP, usual secondary prevention.

Among the changeable risk factors, BMI, DBP and SBP showed no significant difference (BMI: MD –0.16, 95% CI –0.33 to –0.01; *p* = 0.07; *I*^2^ = 38%; Figure 3A. DBP: MD –0.72, 95% CI –1.42 to –0.01; *p* = 0.05; *I*^2^ = 31%; Figure 3B. SBP: MD –2.06, 95% CI –4.24 to –0.11; *p* = 0.06; *I*^2^ = 82%; Figure 3C). However, only the TOSP had significant differences in the field of using the telephone as a remote telemedicine intervention compared with that in USP according to subgroup analysis (BMI: MD –0.87, 95% CI –1.42 to –0.31; *p* = 0.002; *I*^2^ = 0%; Figure 3A. DBP: MD –2.91, 95% CI –4.45 to –1.37; *p* = 0.0002; *I*^2^ = 0%; Figure 3B. SBP: MD –4.09, 95% CI –7.04 to –1.15; *p* = 0.007; *I*^2^ = 60%; Figure 3C). However, it is observed that high heterogeneity existed in SBP. Leave one out sensitivity analysis showed that when we removed three studies respectively, by Dorje et al. (43), Yudi et al. (46), and Hu et al. (39), the overall effect still did not change between the two groups (MD –1.47, 95 CI –3.06–0.12; *p* = 0.07; *I*^2^ = 48).

The results of this meta-analysis indicated that at 10 weeks to 36 months of follow-up there was no significant difference in LDL - C between TOSP and USP [12 studies (14, 19, 27, 30, 32, 33, 36, 39, 41, 43, 46, 50), MD –0.02, 95% CI –0.22–0.18; *p* = 0.83; *I*^2^ = 94%; Figure 3D], the result of the subgroup analysis also showed that there was no significant difference. However, it is also observed that high heterogeneity existed in studies. Leave one out sensitivity analysis showed that when we removed five studies respectively, by Avila et al. (27), Hu et al. (39), Bae et al. (36), Greving et al. (30) and Wong et al. (41), the overall effect changed into a small but significant difference in favor of TOSP (MD –0.11; 95% CI –0.17 to –0.05; *p* = 0.0002; *I*^2^ = 47%).

No statistically significant difference was found in fasting glucose (MD –0.04, 95% CI –0.15 to –0.23; *p* = 0.68; *I*^2^ = 0%; Supplementary Figure 2A), HbA1c (%) (MD –0.03, 95% CI –0.08 to –0.13; *p* = 0.62; *I*^2^ = 0%; Supplementary Figure 2B). A statistically significant difference was seen in smoking cessation rates (RR 0.74, 95% CI 0.59–0.94; *p* = 0.01; *I*^2^ = 0%; Supplementary Figure 2C), however, USP had a better improvement in smoking cessation rates compared with TOSP.

#### Secondary outcomes

##### Physical activity and exercise

**FIGURE 4:**
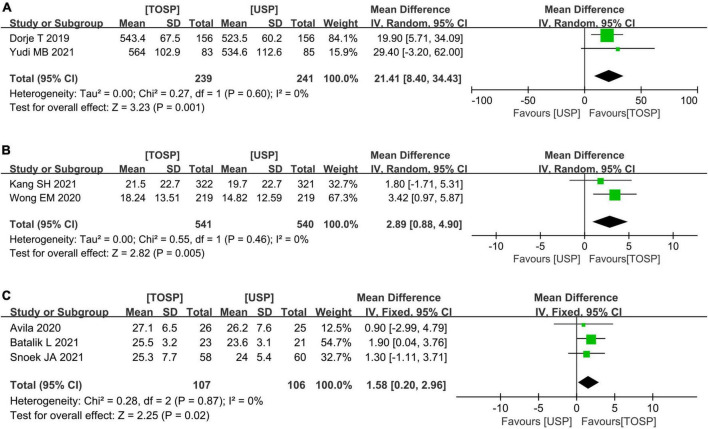
Forest plots of the effects of telemedicine on physical activity and exercise— **(A)** 6MWT; **(B)** GSLTPA score; **(C)** VO_2_ Peak (mL⋅kg^–1^⋅min^–1^); TOSP, telemedicine of secondary prevention; USP, usual secondary prevention.

Fifteen studies (19, 24–28, 32, 33, 35, 36, 38, 41, 43, 46, 50) investigated outcomes on exercise capability at 6 weeks to 6 months of follow-up. Between TOSP and USP, a statistically significant difference was seen in 6 min walk test (6MWT) (MD 21.41, 95% CI 8.40–34.43; *p* = 0.001; *I*^2^ = 0%; Figure 4A), Godin-Sheppard Leisure-Time Physical Activity Questionnaire (GSLTPA score) (MD 2.89, 95% CI 0.88–4.90; *p* = 0.005; *I*^2^ = 0%; Figure 4B), peak oxygen consumption (VO_2_ Peak, mL⋅kg^–1^⋅min^–1^) (MD 1.58, 95% CI 0.20–2.96; *p* = 0.02; *I*^2^ = 0%; Figure 4C).

No statistically significant difference was detected among workload peak (Watt) (MD 15.11, 95% CI –2.05 to 32.28; *p* = 0.08; *I*^2^ = 0%; Supplementary Figure 3A), rating of perceived exertion [RPE (Borg)] (MD –0.18, 95% CI –0.63–0.27; *p* = 0.43; *I*^2^ = 0%; Supplementary Figure 3B), Peak heart rate (Peak HR) (MD 0.00, 95% CI –0.03–0.03, *p* = 0.87; *I*^2^ = 0%; Supplementary Figure 3C), VO_2_ Peak (ml/min) (MD 99.67, 95% CI –8.48–207.83; *p* = 0.07; *I*^2^ = 0%; Supplementary Figure 3D)and peak respiratory exchange ratio (Peak RER) (MD 0.00, 95% CI –0.03–0.03, *p* = 0.87; *I*^2^ = 0%; Supplementary Figure 3E).

##### Muscle function

Avila et al. (27) found that no statistically significant difference was seen in muscle function including sit-to-stand test (*p* = 0.22), isometric quadriceps extension (*p* = 0.33), isokinetic total work (*p* = 0.77), and handgrip strength in the two groups (*p* = 0.49). Nevertheless, Foccardi et al. (38) found that there was a statistically significant difference in the 30 s sit-to-stand test (*p* = 0.03) between TOSP and USP.

##### Exercise compliance

A study reported on exercise compliance. Pandey et al. (24) reported that the mean days of exercise per month and mean hours of exercise per month at the 12th-month follow-up of TOSP had an additional from 4.2 to 17.2 days (*P* < 0.001) and 4.0 h (*P* < 0.001) respectively, compared with USP.

##### Medication adherence

Dorje et al. (43) reported that at the 2nd (*p* = 0.0048), 6th (*p* = 0.019), and 12-month (*p* = 0.011) follow-up, the participants of TOSP tend to be adherent to all four core cardioprotective medications. In addition, Pandey et al. (24) reported on medication adherence (the percentage of days covered), the Figure for participants who were reminded by text messages had a mean 14.2 percentage point improvement compared with USP (*p* < 0.001).

##### Healthy diet

Ögmundsdóttir Michelsen et al. (32) found that the Healthy diet index (*p* = 0.03) differed significantly between patients with MI who were intervened by mobile applications and the patients who received usual care. Hu et al. (39) reported that the patients who received monitoring and follow-up by telephone achieved significantly better dietary control (*p* = 0.0001). Besides, Vale et al. (48) found that patients in the field of intake of fat (*p* = 0.04) and fiber (*p* = 0.02) intervened by telephone differ significantly from the patients in USP. While Maddison et al. (49) and Pfaeffli Dale et al. (50) found that there is no statistically significant difference in fruit and vegetable intake of patients who intervened by sending text messages (*p* > 0.05).

#### Tertiary outcomes

##### Depression and anxiety

Four studies (33, 46, 47, 50) were performed by Hospital Anxiety and Depression Scale (HADS). The data of HADS Anxiety (HADS-A) and HADS Depression (HADS-D) from 6 months to 13 months follow-up showed that no difference was noted between USP and TOSP. (HADS-A: MD –0.03, 95% CI –1.37–1.31; *p* = 0.97; *I*^2^ = 80%. HADS-D: MD –0.21, 95% CI –0.73–0.30; *p* = 0.42; *I*^2^ = 0%; Supplementary Figures 4A,B). However, there was high heterogeneity across studies. Leave one out sensitivity analysis showed that when we removed the study by Pfaeffli Dale et al. (50) the overall effect changed into a small but statistically significant difference in favor of TOSP (MD –0.73; 95% CI –1.42–0.04; *P* = 0.04, *I*^2^ = 0%). In addition, no statistically significant difference was detected in the Depression Anxiety Stress Scales (40) (DASS) (*p* = 0.90) and the Generalized Anxiety Disorder 7-item scale (43) (*p* = 0.17).

##### Self-efficacy

Self-efficacy was reported in 4 studies (22, 40, 41, 50). Due to the different selection of indicators and expressions, it cannot be included in the meta-analysis. Therefore, A narrative synthesis is carried out. Two studies (22, 40) reported Cardiac Self-Efficacy Scale (CSE Scale). Su and Yu (40) found that the improvement of self-efficacy in TOSP was significantly higher than that in USP at 12 weeks (*P* < 0.001), but there was no significant difference in self-efficacy at 6 weeks (*p* = 0.402). Ross et al. (22) showed that there was no significant difference except in the self-efficacy domain (Total plus), where the improvement in self-efficacy of the telemedicine group was worse than that of USP (Control symptoms: *p* = 0.10; Maintain function: *p* = 0.05; Total: *p* = 0.05; Total plus: *p* = 0.03). Wong et al. (41) found that in the terms of Self-efficacy for Exercise (SEE), TOSP showed no improvement at either 6 months or 12 months (6 months: *P* = 0.17;12 months: *p* = 0.90). Pfaeffli Dale et al. (50) reported that no statistically significant difference was noted in the overall self-efficacy for TOSP at 6 months of follow-up (*p* = 0.73).

##### Knowledge score

Three trials reported data on the knowledge score of ACS or CHD. The SMD was used because of the differences in measurement scales. Meta-analysis of the included trials did not show a significant difference in knowledge score between TOSP and USP (SMD 0.38; 95% CI –0.25–1.00; *P* = 0.23, *I*^2^ = 94%). It is observed that high heterogeneity existed in the Knowledge score. Leave one out sensitivity analysis showed that when we removed one study by Dorje et al. (43), the overall effect still did not change between the two groups (SMD = 0.05; 95% CI –0.10–0.20; *P* = 0.53, *I*^2^ = 0%).

##### Economy

Two studies (30, 45) have reported the cost of secondary prevention. Brouwers et al. (45) found that after the 1-year follow-up, overall heart health care costs of TOSP compared with USP were reduced by 40 €, but no significant difference was noted statistically (*p* = 0.36). The study (30) showed that after the 1-year follow-up, patients in the TOSP group and USP group underwent the same health benefits, namely the QALY gain of 0.86–0.85, and the less cost of TOSP than USP of 219 € (95% CI: –2301€ to 1825 €).

##### Safety endpoints

4 studies (13, 22, 26, 37) reported that TOSP and USP in the 2–12 month had no statistical significant differences in all-cause hospitalization (RR 0.89, 95% CI 0.70–1.13; *p* = 0.34; *I*^2^ = 0%, Supplementary Figure 5A). Three studies (13, 22, 37) of cardiac-related hospitalization showed that there is no significant difference between TOSP and USP in the 2–12 months (RR 0.86, 95% CI 0.55–1.36; *p* = 0.53; *I*^2^ = 0%, Supplementary Figure 5B). Meta-analysis of 3 studies (30, 33, 34) about major adverse cardiovascular events (MACE) showed that TOSP and USP in years 1 to 13 months of follow-up study showed no statistical difference (RR 0.73, 95% CI 0.50–1.08; *p* = 0.12; *I*^2^ = 0%, Supplementary Figure 5C). Similarly, Bermon et al. (13) and Treskes et al. (34) comparing the effect of TOSP and USP, did not find statistical differences between all-cause mortality and cardiac-related mortality.

##### Assessment of publication bias

We explored publication bias using funnel plots for the primary outcomes: (1) BMI; (2) SBP; (3) DBP); (4) low-density lipoprotein cholesterol (LDL-C). Funnel plots were shown in Supplementary Figure 6. Symmetry was observed in the BMI and DBP. However, there is dissymmetry observed in the funnel plots of SBP and LDL-C. Publication bias may exist in the meta-analysis.

## Discussion

32 RCTs were included in our meta-analysis. We compared the outcomes of the patients with ASCVD who received secondary prevention in TOSP and those in USP. In this systematic review, we found that telemedical interventions have successfully been implemented for the improvement of some risk factors, physical activity, and exercise. No statistically significant difference was noted in blood lipids, blood glucose, depression and anxiety, and safety endpoints. It remained controversial in muscle function, medication adherence, exercise compliance, knowledge score, healthy diet, self-efficacy, and economy.

We found some evidence of differences in the primary outcomes of TOSP compared to USP under different interventions (mobile application, telephone, text message, website, website, and mobile application). When the telephone was used as an main intervention, significantly favorable changes in BMI, SPB, DBP. However, a Meta-analysis for blood lipids and smoking cessation did not confirm better results for TOSP than for USP. Huang et al. (51) reported that there was no difference in changeable risk factors (blood lipids, blood pressure, smoking, and weight) between patients with telemedical intervention and those with center-based cardiac rehabilitation in the short-term (12 weeks to 12 months) and long-term (up to 6 years) follow-up. However, it is reported by Turan Kavradim et al. (52) that telemedical intervention was associated with an increase in Waist circumference, total cholesterol (TC), and triglycerides when compared with the usual care group. In a meta-analysis (53), it is observed that significant improvements existed in the TC, SBP, and smoking in patients who received the telemedical intervention. The reasons might be as follows: (1) The inclusion of patients in our study included all ASCVD diseases, which is wider than the range of CHD included in previous systematic reviews. (2) Compared with previous systematic reviews, this study was analyzed with interventions as subgroups. In addition, Turan Kavradim et al. (52) reported that using the telephone as an intervention is the most popular telemedical intervention. Klimis et al. (54) also found that text messages were more effective compared with mobile applications in the systematic review. Huang et al. (51) reported that telephone-based interventions have the greatest evidence value for secondary prevention. Our systematic review also showed that telephone-based intervention was more effective in reducing risk factors. The reasons might be (1) the majority of ASCVD patients are elderly, but the elderly use smartphones, apps, and websites rarely; (2) successful contact by telephone is better than that by text messages. Telemedicine is an acceptable and appropriate method to improve the coverage and utilization of secondary prevention. However, pragmatic implementation studies are needed to realize the impact of telemedicine on the accessibility of secondary prevention.

We found that a statistically significant MD of 1.58 mL⋅kg^–1^⋅min^–1^ in the VO_2_ Peak (mL⋅kg^–1^⋅min^–1^) was noted between TOSP and USP. In the early study, a 1 mL⋅kg^–1^ ⋅min^–1^ increase of VO_2_ Peak (mL⋅kg^–1^⋅min^–1^) can lead to a 10% decrease in Cardiovascular mortality (55), and it can be regarded as an important indicator of morbidity and mortality in patients with CVD (56). Nevertheless, Sumeet Gandhi et al. (57) reported that there is no statistically significant difference in the terms of VO_2_ Peak (mL⋅kg^–1^⋅min^–1^) between the e-health group and the control group. We offer some possible explanations for the inconsistency as follows: the inconsistency of duration, intensity, frequency, and engagement of intervention, which may affect the action and effectiveness of interventions. Therefore, it is important to explore an effective and accessible intervention method. Besides, it’s necessary to have more evidence to assess the outcomes. We also found that in terms of subjective measures (GSLTPA score), TOSP had improvement in physical activity compared with USP while no statistically significant difference was noted for other objective measures [RPE (BORG),Peak HR, VO_2_ Peak (mL/min),Peak RER]. The reason probably is few studies analyze the indicators. Therefore, firmer conclusions need more research.

In the terms of muscle function, we made a narrative synthesis for the two included studies. Due to the selection of different indicators, it is difficult to measure the effect of these outcomes and confirm the outcomes by meta-analysis. A 24-year follow-up study including 1 142 599 participants shows that low muscle strength is associated with all-cause and cardiovascular mortality in CVD (58). Similarly, in a population-based prospective cohort study, people with higher grip strength had a lower risk of all-cause mortality and CVD morbidity and mortality (59). However, at present, it is unclear whether telemedical interventions make a difference in muscle strength in patients with ASCVD. It is a prospective topic in future studies.

A narrative synthesis was done in the area of a healthy diet. It is difficult to make definitive conclusions in this section because of the limited number of included studies and the different indicators in each study. The guidelines recommend a healthy diet as a cornerstone of CVD prevention and suggest a Mediterranean diet or a similar diet for ASCVD patients. The Mediterranean diet includes high intakes of fruits, vegetables, legumes, whole grain products, fish and olive oil, moderate alcohol consumption, and small amounts of (red) meat, dairy products, and saturated fatty acids (1). A meta-analysis showed that better adherence to the Mediterranean diet was significantly associated with a 10% reduction in cardiovascular morbidity or mortality and an 8% reduction in all-cause mortality (60). A multicenter study that included 7,447 subjects showed that a nut-rich Mediterranean diet reduced the risk of ASCVD by 28% and a diet rich in olive oil by 31% (61). Therefore, a healthy diet is necessary for the secondary prevention of ASCVD (62). At present, it is inconclusive whether telemedicine can have a beneficial effect on the healthy diet of ASCVD patients. This could be a focus of future research in telemedicine.

In the system review, there was no statistically significant difference in knowledge score. There is a gap between knowledge of CVD conditions and their risk factors in patients with CVD, which are important barriers between the effective prevention and treatment of CVD (63). Kim et al. reported that specialized interventions should focus on groups with less knowledge of CVD (64). Because of educational needs, it is a good choice to use telemedicine to learn knowledge of ASCVD.

A study reported that self-efficacy, a robust predictor of behavioral persistence, should be scheduled to improve exercise adherence (65). Besides, general and exercise-specific self-efficacy are relevant to the quality of life (66). Therefore, the improvement of self-efficacy supported by telemedicine is worth exploring. In the adherence part of secondary prevention through telemedicine, we classified adherence as medication adherence, and exercise compliance. In our systematic review, both the subjective and objective indicators showed that telemedicine can improve medication adherence and exercise adherence to some extent. In the economic part, we found that the patients in the telemedicine group paid less tuition for secondary prevention compared with the usual care group, but not significantly. In this systematic review, the adherence part and the economics part are both descriptive analyses, and the conclusions drawn may be one-sided.

Limitations: (1) There was a difference in the follow-up time of RCTs included in our studies. Here, we choose the outcomes with the longest follow-up time of RCTs. Therefore, inconsistency of research time existed in our study. (2) Due to a limited number of studies, it is impossible to determine which intervention is more effective for some indicators. (3) Because of the intervention methods included in RCTs, it is difficult to blind participants in studies. Therefore, the third item of assessing the risk of bias (blinding of participants and personnel) cannot be avoided. (4) Few studies included patients with ischemic stroke and PAD who received secondary prevention through telemedicine. It is found that secondary prevention supported by telemedicine may be probably effective in cerebrovascular disease and PAD (67, 68). However, existing studies often lack adequate patients, standardized endpoints, and confirmation of independent researchers. Therefore, a larger scale with standardized powerful trials of endpoints is worth exploring.

## Conclusion

The meta-analysis shows that there is a net benefit of secondary prevention supported by telemedicine (especially when using the telephone as an intervention) in patients with ASCVD in the terms of some risk factors, physical activity and exercise. There are still controversies to improve medication adherence, exercise compliance, muscle function, knowledge score, and self-efficacy *via* telemedicine, which is worth exploring. Larger samples size and longer-term follow-ups are needed in future studies.

## Data availability statement

The original contributions presented in this study are included in the article/Supplementary material, further inquiries can be directed to the corresponding author/s.

## Author contributions

HJT, LXW, and BLZ designed the research project. LYD, QW, and FD performed the studies selection and quality evaluation of included studies. LYD, QW, FD, and YFL analyzed the data and wrote the manuscript. HJT revised the manuscript. All authors contributed to the preparation of the manuscript and agreed to be accountable for all aspects of the work ensuring integrity and accuracy.
