# Supplementary material for: The effect of telemedicine on secondary prevention of atherosclerotic cardiovascular disease: A systematic review and meta-analysis
Source: Front Cardiovasc Med. 2022 Nov 3;9:1020744. doi: 10.3389/fcvm.2022.1020744 (PMC9683074; doi:10.3389/fcvm.2022.1020744)
Supplement: Supplementary file 1 [file Data_Sheet_1.docx]

Supplementary Material

# Supplementary Tables and Figures

## Supplementary Tables

### Supplementary Table 1: Search Strategy

We searched PubMed and Web of Science, CINAHL, EBSCO, MEDLINE, embase, and Cochrane Library (May 2022), updating from the construction of the bank to May 2022.

| Patient populations | #1 | "Cardiovascular disease" OR "Heart Disease*" OR "Acute Coronary Syndrome*" OR "Coronary Artery Disease" OR "Myocardial Ischemia" OR "Coronary Artery Disease*" OR "Ischemic Heart Disease" OR "Peripheral artery disease" |
| --- | --- | --- |
|  | #2 | "myocardial infarction" OR "Angina Pectoris" OR "Coronary Artery Bypass" OR "percutaneous coronary intervention" OR "Percutaneous Coronary Revascularization" OR "Percutaneous Transluminal Coronary Angioplast*" OR "Coronary Balloon Angioplast*" OR "atherosclerotic cardiovascular disease" OR "Arterial occlusion" OR "Ischemic Stroke" OR "peripheral arteriosclerosis obliterations" |
|  | #3 | #1 OR #2 |
| Intervention | #4 | "telemedicine" OR "videoconferencing" OR "teleconferenc*" OR "meeting" OR "telemonitor*" OR "teleconsultation" OR "teleeducacion" OR "Telenursing" OR "telecare" OR "telerehabilitation" OR "tele-rehabilitation" OR "Remote Rehabilitation" OR "Virtual Rehabilitation" OR "telehone" OR "tele-home" |
|  | #5 | "mobile communication*" OR "mobile health" OR "mealth" OR "m-health" OR "mobile device" OR "telehealth" OR "tele-health" OR "eheat" OR "e-health" OR "digital health" OR "mobile technolog*" OR "website" OR "Internet" OR "mobile apps" OR "smartphone application" OR "mobile application" OR "mobile phone" OR "smartphone" OR "smart phone" OR "telephone" OR "cellphone" OR "cellular phone" OR "text messaging" OR "message" |
|  | #6 | #4 OR #5 |
|  | #7 | "secondary prevention" OR "secondary level of prevention" OR "Cardiac rehabilitation" OR "cardiac rehabilitation" OR "Rehabilitation" OR "rehab" |
|  | #8 | "revascularisation" OR "medication" OR "dietary intervention" OR "physical activity" OR "smoking cessation" OR "rehabilitation" OR "psychosocial support" OR "counseling" |
|  | #9 | #7 OR #8 |
| Combined | #10 | #3 AND #6 AND #9 |

## Supplementary Figures

### Supplementary Figure 1


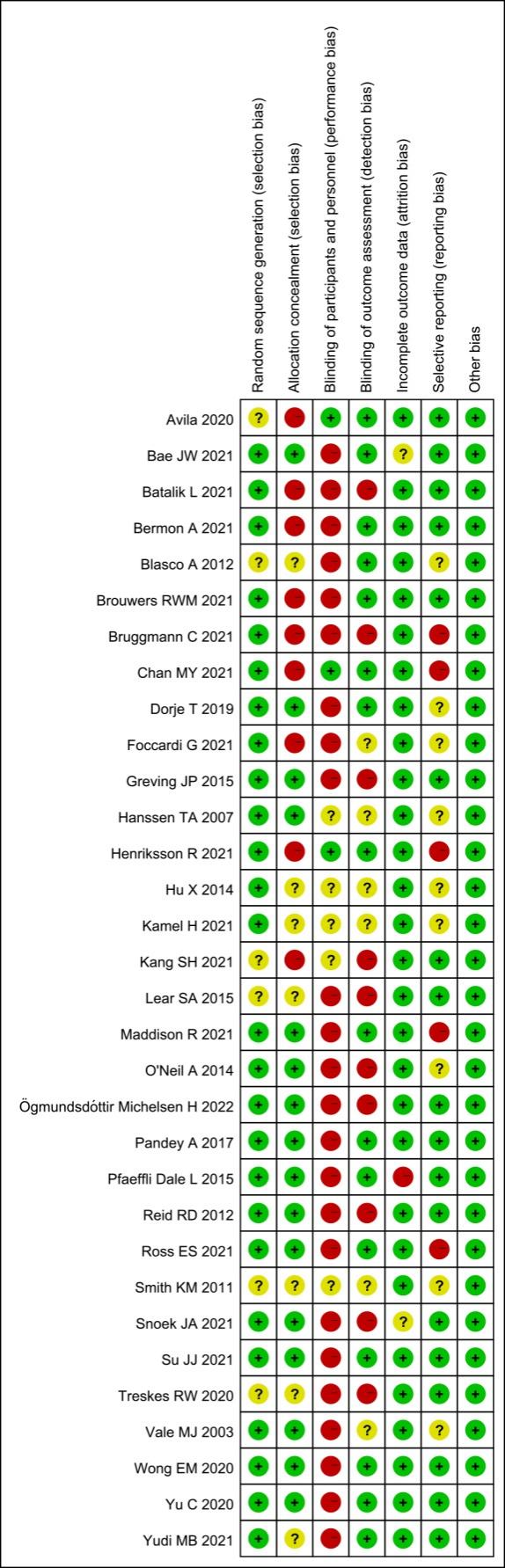


Supplementary Figure 1 Summary of the results of the risk of bias

### Supplementary Figure 2

| (A) |
| --- |
| 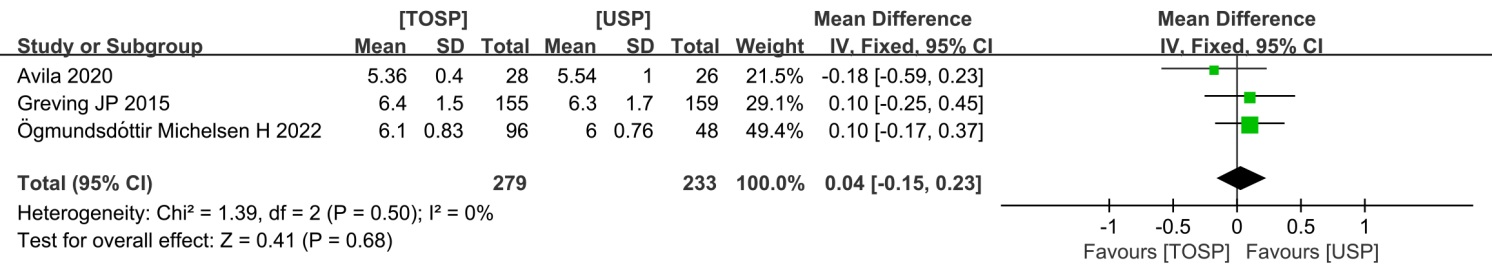 |
| (B) |
| 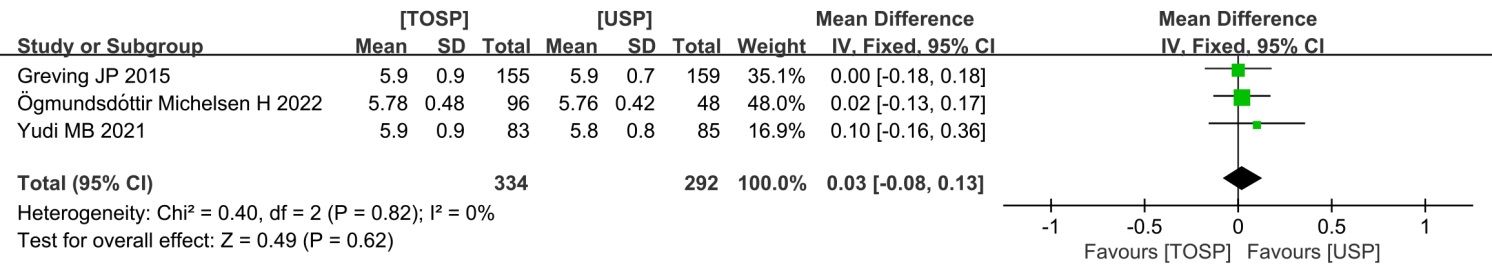 |
| (C) |
| 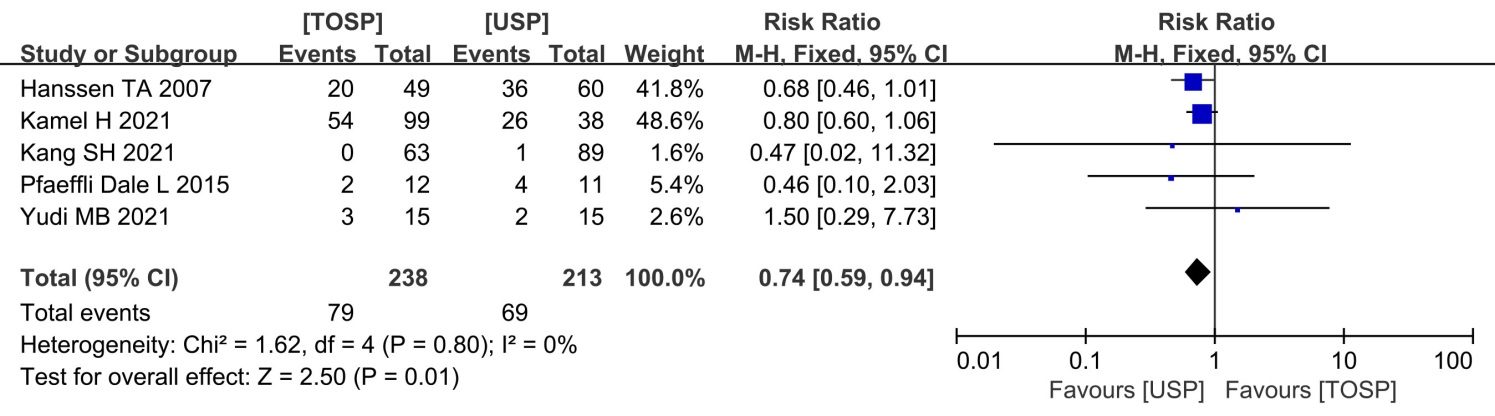 |

**Supplementary Figure 2**：Forest plots of the effects of telemedicine on risk factors—(A) fasting glucose; (B) HbA1c(%); (C) smoking cessation rates; TOSP, telemedicine of secondary prevention; USP, usual secondary prevention.

### Supplementary figure 3

| (A) |
| --- |
| 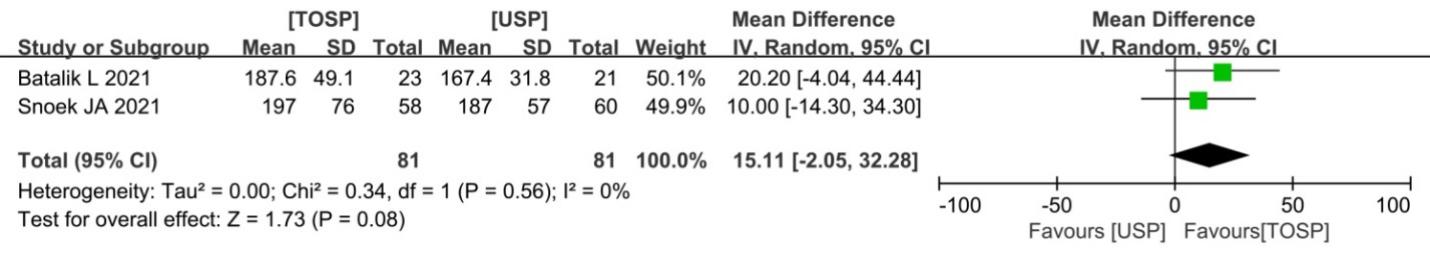 |
| (B) |
| 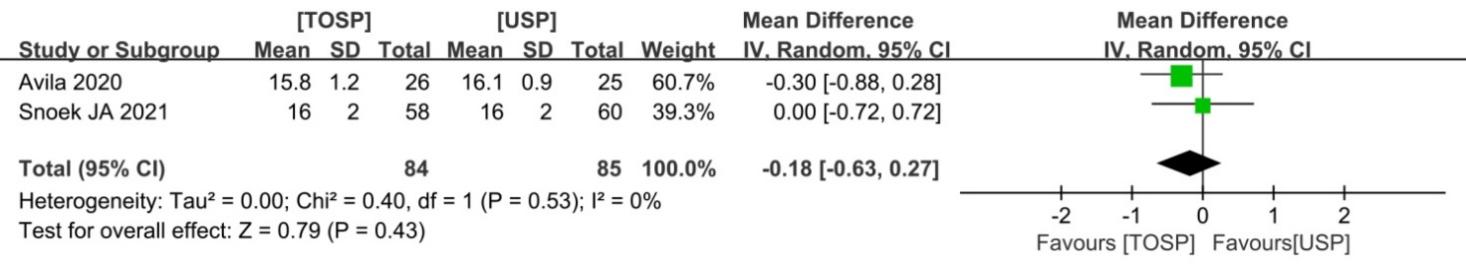 |
| (C) |
| 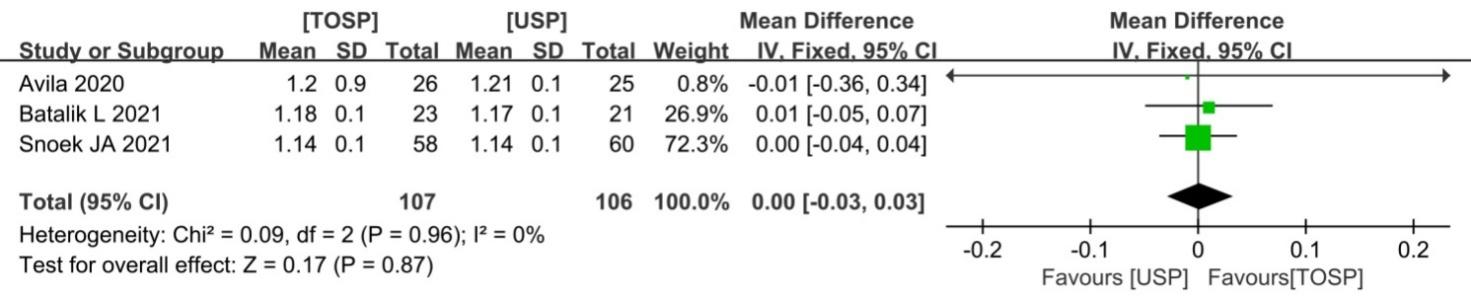 |
| (D) |
| 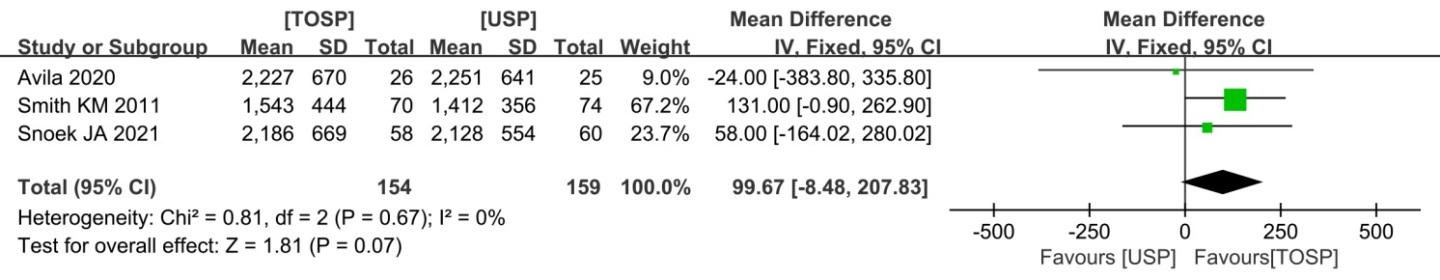 |
| (E) |
| **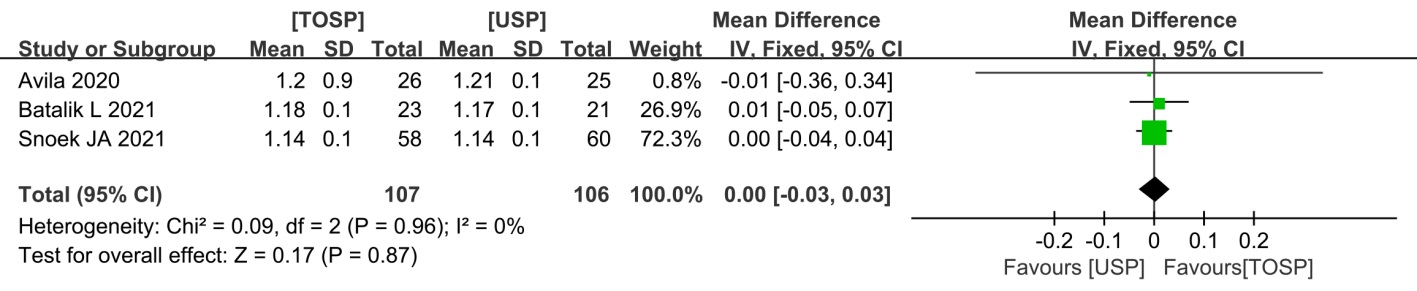** |

**Supplementary figure 3**：Forest plots of the effects of telemedicine on physical activity and exercise—(A) workload peak(Watt); (B) RPE (Borg); (C) Peak HR ; (D) VO2 Peak (ml/min) (E) Peak RER; TOSP, telemedicine of secondary prevention; USP, usual secondary prevention.

### Supplementary figure 4

| (A) |
| --- |
| 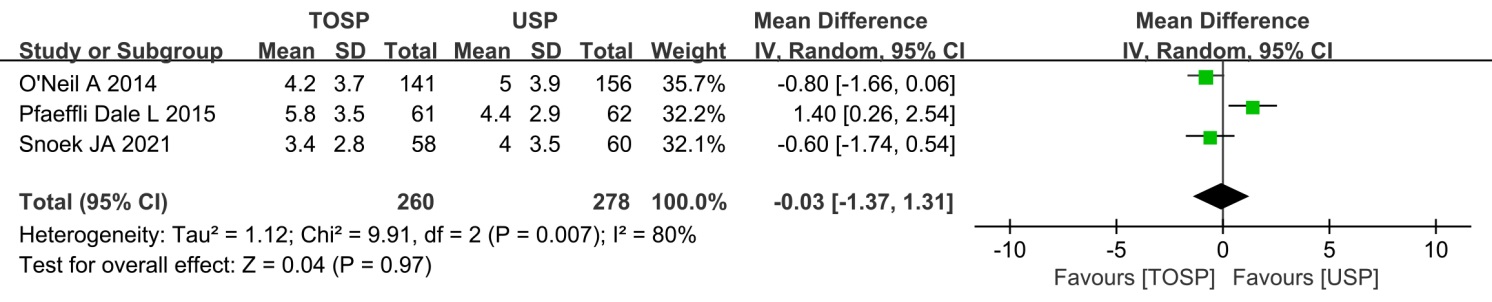 |
| (B) |
| 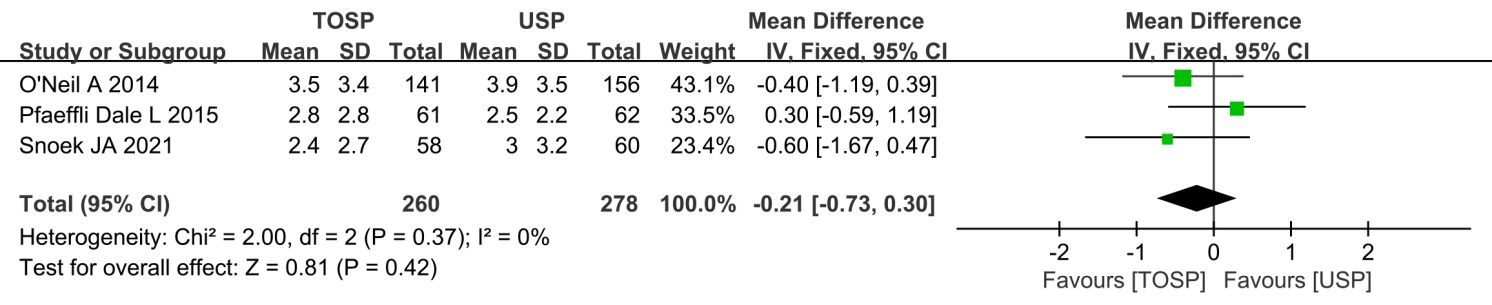 |

**Supplementary figure 4**：Forest plots of the effects of telemedicine on depression and anxiety—(A) HADS-A; (B) HADS-D; TOSP； telemedicine of secondary prevention; USP, usual secondary prevention

### Supplementary figure 5

| (A) |
| --- |
| 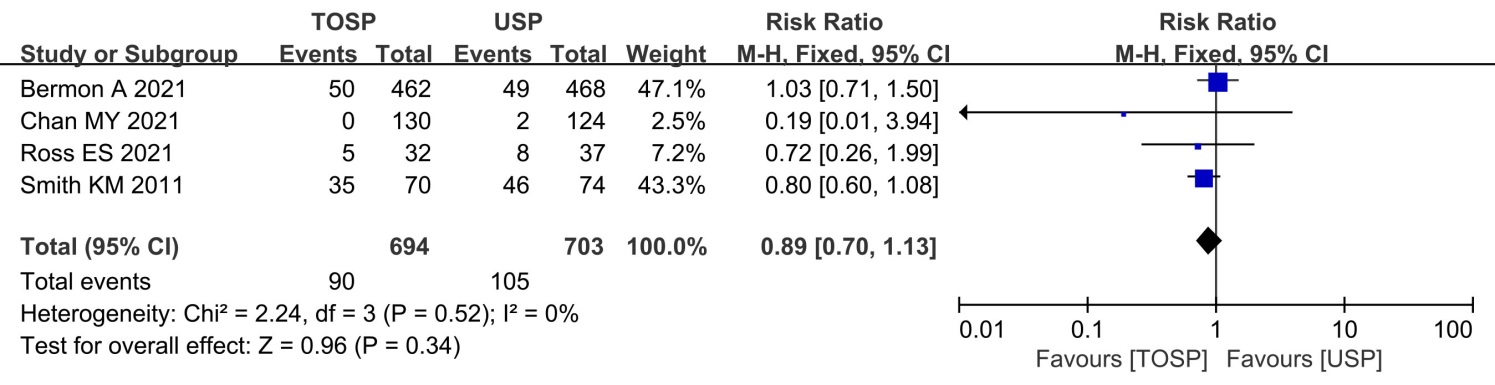 |
| (B) |
| 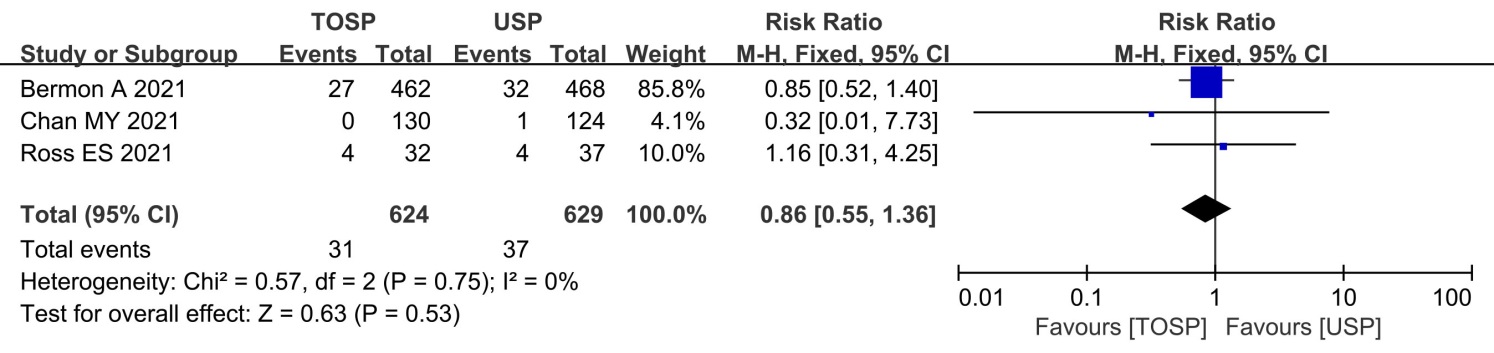 |
| (C) |
| 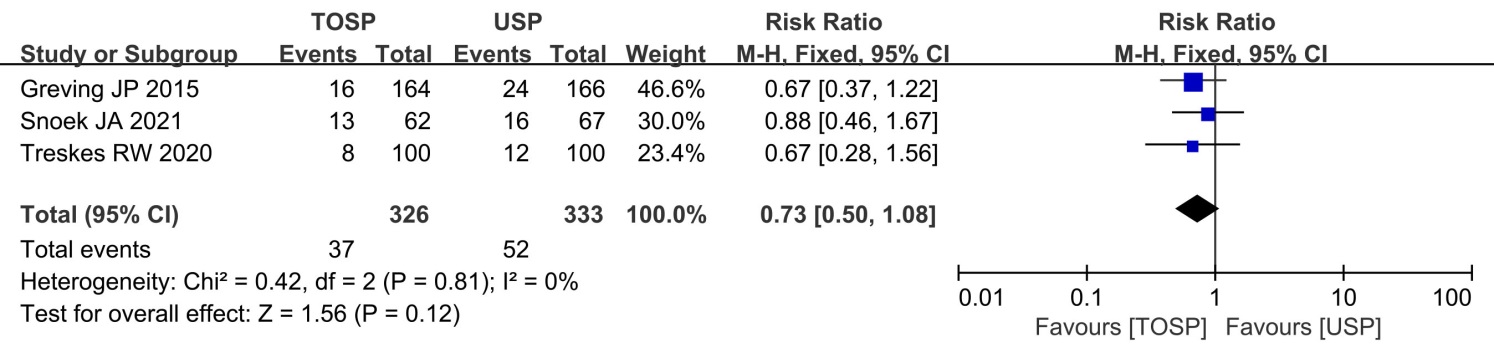 |

**Supplementary figure 5**：Forest plots of the effects of telemedicine on safety endpoints—(A) all-cause hospitalization; (B) cardiac-related hospitalization; (C) MACE, telemedicine of secondary prevention; USP, usual secondary prevention.

### Supplementary figure 6

| (A) | (B) |
| --- | --- |
| 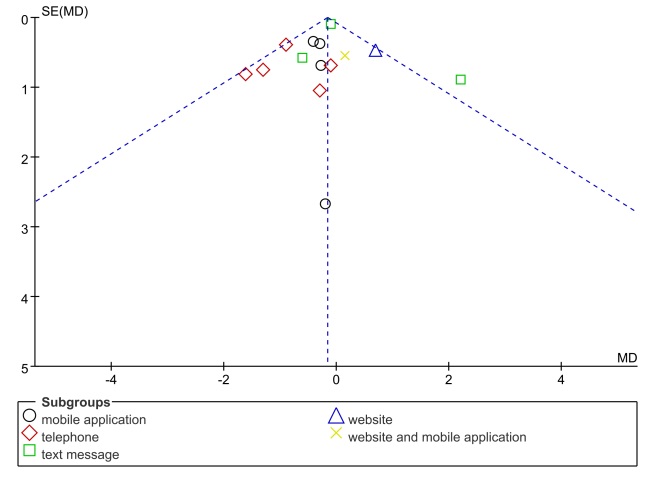 | 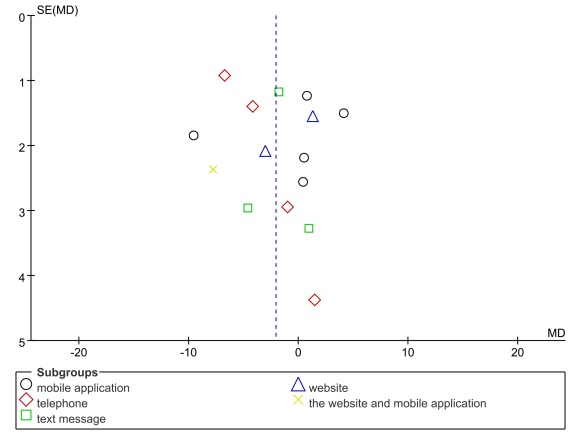 |
| (C) | (D) |
| 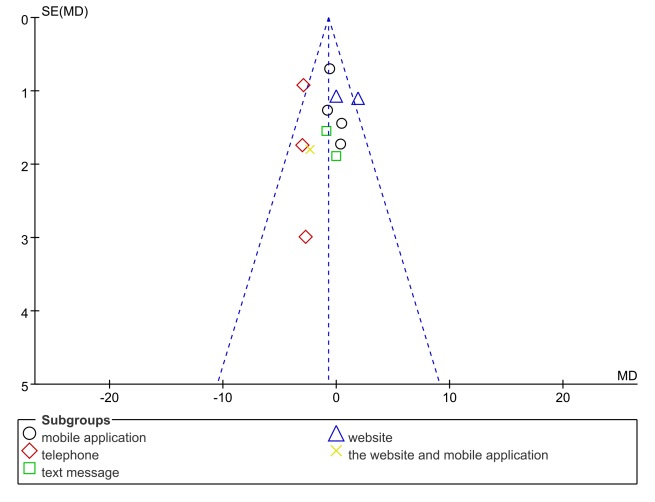 | 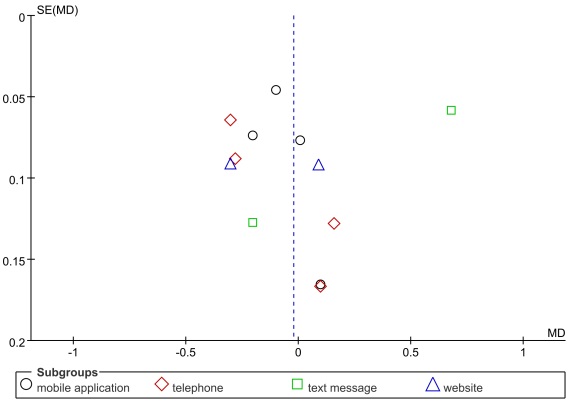 |

Supplementary figure 6：Funnel plot in the primary outcomes: (A) BMI; (B) SBP; (C)DBP; (D) LDL-C
